# Erector Spinae Plane Block versus Quadratus Lumborum Block for Postoperative Analgesia after Laparoscopic Resection of Colorectal Cancer: A Prospective Randomized Study

**DOI:** 10.1155/2024/6200915

**Published:** 2024-03-18

**Authors:** Dina Mahmoud Fakhry, Hatem ElMoutaz Mahmoud, Dina Yehia Kassim, Hebatallah NegmEldeen AbdElAzeem

**Affiliations:** Department of Anesthesiology, Surgical Intensive Care and Pain Management, Faculty of Medicine, Beni-Suef University, Beni-Suef, Egypt

## Abstract

**Background:**

In recent years, the attention paid to colorectal cancer (CRC) surgery and postoperative analgesia has increased.

**Objective:**

The objective of the current study was to compare the impact of ultrasound-guided erector spinae plane block (ESPB) and transmuscular quadratus lumborum block (TQLB) upon providing relief to patients with postoperative pain who underwent laparoscopic resection for CRC.

**Methods:**

In this prospective, comparative, and randomized study, the authors considered a total of 60 patients who chose to undergo laparoscopic resection for colorectal cancer. The total number of patients was randomly divided into two groups (such as ESPB and TQLB) so that each group had a total of 30 patients. For the former group, i.e., the ESPB group, 20 ml of 0.25% bupivacaine was administered at each side for bilateral ultrasound-guided erector spinae plane block, while the latter group received the same dose of medicine for bilateral ultrasound-guided transmuscular quadratus lumborum block (TQLB). The researchers recorded the first time to rescue an analgesic, the whole amount of rescue analgesia under consumption in the first 24 hours after the surgical procedure, and associated adverse events.

**Results:**

Among the groups considered, the ESPB group took a significantly lengthy time to raise a first request for rescue analgesic (280 ± 15.5 min) in comparison with the TQLB group (260 ± 13.8 min). Likewise, the consumption of overall nalbuphine was remarkably lesser in the ESPB group during the first 24 hours (24 ± 2.5 mg) compared to the TQLB group (30.5 ± 1.55 mg).

**Conclusion:**

The analgesic efficacy of ESPB was better when compared to TQLB in terms of time to rescue analgesia and overall opioid consumption during the first 24 hours. This study was registered at ClinicalTrials.gov on 10/10/2022 (registration number: NCT05574283).

## 1. Background

In recent years, there has been a tremendous increase in the incidence of colorectal cancer (CRC), and the disease has become one of the top causes of cancer-related deaths [1]. In this background, there has been a surge in recent years to give prime importance to CRC surgical procedures and postoperative analgesia [2]. Laparoscopic surgery is the most commonly applied surgical procedure for curative resection of CRC, and it accounts for up to 60% of the surgical procedures [3]. In spite of the fact that the procedure is less invasive in nature, laparoscopic surgery causes moderate to severe acute postoperative pain [4]. When the postoperative pain control measures are poorly engaged, it extends the hospital stay of the patients and postoperative care while also causing heavy dissatisfaction among the patients [5].

For an established period of time, opioids have been preferred as postoperative analgesics although these drugs were proved earlier to have extreme side effects on patients, such as enteroparalysis, vomiting, and nausea. These reactions remain nonconducive for the patient to recover after the surgery is completed [6]. Epidural anaesthesia has been proved to function as a superior postoperative analgesic with a high quality of recovery (QoR) rate. However, the application of this procedure is highly confined since it induces hypotension most of the time and is complex to apply during the surgical procedure [7]. Forero et al. developed a new interfascial plane block technique called erector spinae plane block (ESPB), in which local anaesthesia is administered under the guidance of ultrasound between the deep fascia of the erector spinae muscle and the vertebral transverse process. This procedure relieves the patient from pain experienced in the thoracoabdominal region [8]. The ultrasound-guided ESPB procedure is easy to perform and can be relied upon. In comparison with transversus abdominis plane (TAP) block, the ESPB technique is advantageous as it provides the benefits of thoracic epidural analgesia (TEA), such as blocking the sensory nerves in the thorax and abdomen [9, 10]. In addition to these, the complications involved in ESPB are too low compared to TEA or paravertebral blocks [11]. The quadratus lumborum block (QLB) is one of the newly found posterior abdominal trunk blocks, and it can generate analgesic effects via local anesthetic. It covers both the thoracic paravertebral space as well as the thoracolumbular fascia. The QLB techniques are of three types, depending on how the injection is positioned and approached, such as lateral, anterior, and posterior [12]. Among these types, the anterior transmuscular quadratus lumborum block (TQLB) is a truncal block (ventral rami of T7-L2), and it generates the analgesic effect by spreading through the splanchnic nerves in the celiac ganglion and by creating a block in the ventral rami of the lower spinal nerves, the thoracic sympathetic trunk, and the sympathetic fibers and mechanoreceptors within the thoracolumbar fascia [13, 14].

In this study, we compared the impact of ultrasound-guided ESPB and TQLB procedures on providing relief to patients with postoperative pain after laparoscopic resection for colorectal cancer. The primary objective was the total dosage of rescue analgesia used in the first 24 hours after the procedure was over. Secondary outcomes included the first time to request for rescue analgesic (min), pain intensity during rest and movement using VAS scores during the first 24 hours after the surgery, QoR, and adverse events.

## 2. Methods

The current study utilized a prospective, randomized, and single-center research design and included 60 patients who underwent elective laparoscopic resection for CRC. The study was conducted at Beni-Suef University Hospital between November 2022 and September 2023. The study was approved by the Ethics Committee, Faculty of Medicine, Beni-Suef University (FM-BSU) under (Identifier: FM-BSU REC/11092022/Negm). Furthermore, the study was registered on ClinicalTrials.gov on 10/10/2022 (registration number: NCT05574283). Participation was voluntary, and informed consent was obtained from subjects prior to participation. The current study adhered to the principles of the Declaration of Helsinki. This study included patients (*n* = 60) of ASA grades I–III of both genders. The age group of the patients was 35–75 and underwent elective laparoscopic resection for CRC. The study's exclusion criteria included (1) patient's refusal to participate in the study, (2) established hypersensitivity to any medication involved in the study, (3) chronic opioid usage or chronic pain patient, (4) liver insufficiency (defined as serum bilirubin ≥34 *μ*mol/l, albumin ≤35 g/dl, and INR ≥1.7), (5) renal insufficiency (termed as glomerular filtration rate <44 ml/min), (6) morbid obesity (defined as a BMI >35), and (7) obstructive sleep apnea syndrome. The total number of patients was equally randomized into two groups, each consisting of 30 patients. The ESPB group received 20 ml of 0.25% bupivacaine for each side of the bilateral ultrasound-guided erector spinae plane block. In contrast, the TQLB group received the same drug and dosage for bilateral ultrasound-guided transmuscular quadratus lumborum block. The samples were randomized using computer-generated random numbers. Subsequently, they were placed in opaque envelopes under the supervision of a data administrator.

### 2.1. Anesthetic Technique

The entire study population (*n* = 60) was made to undergo the usual checkups, such as cardiac evaluation and hematological and biochemical analyses, prior to the operative procedure. During the preoperative evaluation day, all study participants were given an overview of the study protocol and the visual analogue scale (VAS). The VAS is a pain rating scale. Scores are based on self-reported measures of symptoms that are recorded with a single handwritten mark placed at one point along the length of a 10-cm line that represents a continuum between the two ends of the scale—“no pain” on the left end (0 cm) of the scale and the “worst pain” on the right end of the scale (10 cm). Measurements from the starting point (left end) of the scale to the patients' marks are recorded in centimeters and are interpreted as their pain [15]. When the patients were shifted to the operating room, they were monitored as per the usual standards. Then, the catheter was placed in the central venous system of the right jugular internal vein, followed by monitoring the invasive arterial BP. Then, the patients were intravenously administered with 0.05 mg/kg of midazolam and 4 mg of ondansetron, three minutes before the induction. Afterwards, the patients were anesthetized with 1.5–2 mg/kg propofol, 2 *μ*g/kg fentanyl, and 0.5 mg/kg atracurium. The patients were ventilated using a face mask and 100% oxygen was supplied to the patients at a rate of 4 L/min along with 1.2% isoflurane. The patients were intubated after 180 seconds using a cuffed oral tube of appropriate size. Furthermore, 1.2% isoflurane was also maintained at 100% oxygen for anaesthesia, whereas fentanyl was infused intravenously at 1-2 *μ*g/kg/hr. Then, the patients' muscle relaxation was continued using 0.1 mg/kg of atracurium for every 20 min. In order to ensure end-tidal carbon dioxide levels in the range of 35–40 mmHg, all patients were mechanically ventilated. During the operation, the IV fluid requirements were measured and ensured, whereas the body temperature of the patient was maintained to be normal during the entire surgical procedure. Towards the end of the procedure, the muscle relaxant was reversed using neostigmine (0.04 mg/kg) and atropine (0.015 mg/kg). After the extubation got over, the patients were shifted to the post anesthesia care unit (PACU). Once the patients exhibited stable vital points and were fully awake, they were shifted to the surgical intensive care unit.

### 2.2. Intervention

The entire set of blocks considered for the study was performed after the airway was secured, prior to the beginning of the surgical procedure.

#### 2.2.1. Ultrasound-Guided ESPB Procedure

Under aseptic conditions, the researchers performed the ESPB procedure with the guidance of ultrasound. Every patient was maintained in the right lateral decubitus position. Through direct palpation of the spinous processes that begin from C7 downward, the T7 spinous process was identified. Afterwards, the tip of the T7 transverse process was found with the help of a linear array high-frequency ultrasound probe (PHILIPS HD5). This probe was then placed in a transverse orientation. By shifting this probe to a different orientation, i.e., portrait, the researchers captured the parasagittal view of the skin, erector spinae muscle, trapezius, and subcutaneous tissue. The T7 transverse process was confirmed after the disappearance of the rhomboid muscle since the latter is present at T5-T6 vertebrae. In order to make sure that the needle has been inserted correctly, a 22-G 80 mm needle (Pajunk SonoPlex® STIM; Geisingen, Germany) was inserted in plane craniocaudally until it reached the T7 transverse process. Then, the researchers injected 0.5–1.0 mL of saline. Once the saline was injected, its distribution was evaluated so that factors such as the injection plane and perfect needle position could be verified. Since there was no distension present in the erector spinae muscle, it was confirmed that the needle tip reached the correct plane. Then, the patients were bilaterally administered with 20 mL of 0.25% bupivacaine.

#### 2.2.2. Ultrasound-Guided TQLB Procedure

After making the patient lay in the right lateral decubitus position, a low-frequency curvilinear ultrasound transducer (PHILIPS HD5) was placed on the patient under aseptic conditions. This transducer was placed above the anterior and posterior iliac crest and below the rib cage. The transducer was medially moved until the quadratus lumborum (QL) muscle was identified at its place of attachment on the lateral edge of the transverse process of the 4th lumbar vertebra. At the anterior position, the psoas major muscle was found, whereas at the posterior end, the erector spinae muscle was found with its respective attachment at the transverse process. Then, a 22-G 80 mm needle (Pajunk SonoPlex® STIM; Geisingen, Germany) was inserted in the plane of the transducer (lateral edge) and progressed it through the quadratus lumborum muscle until it passed through the ventral fascia propria of the QL muscle. The position of the needle was confirmed by injecting 5 ml of normal saline (0.9%) followed by the visualization of hydrodissection. Once the aspiration was done, 0.25% bupivacaine (volume 20 ml) was administered in parallel with visualization of the psoas muscle compression. For the other side too, the same process was repeated.

Following is the list of parameters recorded during the investigation:

- Characteristics of the patient: age, gender, BMI, and ASA physical status.
- Time required to execute the technique (in minutes): this measure is defined as the time required to perform the ultrasonic visualization, introduction of the needle, and drug injection in an appropriate manner (i.e., the time taken from the placement of the ultrasound probe on the patient's skin to the conclusion of the local anesthetic injection) [16].
- Duration of anesthesia, surgery, and PACU stay.
- Vital parameters such as the heart rate (HR) and mean arterial blood pressure (MAP) were measured at their baseline values prior to anesthesia administration. Then, the patients were continuously monitored, and their vital scores were recorded every 10 minutes intraoperatively.
- Visual Analogue Scale (VAS) [17]: this score was calculated at rest as well as at the time of movement at 30 minutes, followed by 1, 3, 6, 12, and 24 hours postoperatively. For pain relief, if the score is ≤3, it is considered to be an acceptable limit. Nalbuphine was intravenously administered (0.15 mg/kg) as an additional rescue analgesia when the VAS was ≥4.
- First time for rescue analgesic (min): this measure denotes the time to raise the first request for postoperative analgesia (nalbuphine) and was calculated after the operation was over to the time when the patient reports VAS ≥4.
- Overall dosage of rescue analgesia (nalbuphine): this value shows the overall dosage consumed in the first 24 hours after surgery.
- Quality of recovery (QoR): this measure uses a 15-questionnaire score for 24 hours after the procedure was completed, and the questionnaire is based on the Korean version of the QoR-15 scale [18]. It contains two components, such as mental and physical well-being [19].
- The researcher recorded the intraoperative and postoperative complication values related to blocks such as lower limb weakness, retroperitoneal hematoma, injury caused by the needle upon vital organs, local anaesthetic toxicity, and hypotension.
- The study also recorded other critical values, such as the incidence and severity of postoperative complications such as sedation, vomiting, respiratory depression, hypotension, bradycardia, and nausea during the first 24 hours of the surgical procedure. The study used a categorical scoring system (0 = none, 1 = nausea, 2 = retching, and 3 = vomiting) to determine nausea and vomiting [20]. The sedation scale (0 = awake, 1 = drowsy, 2 = asleep but arousable, and 3 = deeply asleep) was used to calculate the sedation scores. If the patients secure a sedation score of >0 at any time during the first 24 hours after surgery [21], then they are categorized as sedated. Patient satisfaction was determined using four scales (1 = poor, 2 = moderate, 3 = good, and 4 = excellent) [22].

The primary outcome of the study was the total dosage of rescue analgesia (nalbuphine) used in the first 24 hours after the procedure was over. The secondary outcomes of the study were the first time to request for rescue analgesic (min), pain intensity during rest and movement using VAS scores during the first 24 hours after the surgery, QoR, and adverse events.

### 2.3. Sample Size

The sample size for the study was calculated by comparing the total dosage of rescue analgesia needed between the patients who underwent laparoscopic resection for CRC, treated with ESPB and TQLB. As per the literature [23], the mean ± SD of the total rescue analgesia dosage in the erector spine group was approximately 190.5 ± 34 mg, while in the case of the quadratus lumborum group, it was approximately 159 ± 40 mg. In line with this, the researchers finalized the minimum appropriate sample size to be 26 each in both groups so that the null hypothesis can be rejected at 80% power, i.e., *α* = 0.05 level using Student's *t*-test for independent samples. PS Power and sample size calculation software (v3.1.2) for MS Windows (William D. Dupont and Walton D., Vanderbilt University, Nashville, Tennessee, USA) were used to determine the final sample size. As per the calculations, the final sample size was determined to be 30 patients in each group, considering the dropout.

### 2.4. Statistical Analysis

The numerical data that got normally distributed are shown in the form of the mean ± standard deviation (±SD) whereas the abnormally-distributed data are shown as the median and range or interquartile range (IQR). Furthermore, the qualitative data, i.e., categorical ones, are shown in the form of frequencies (number of cases) and percentage. The numerical data were analysed for normal assumption with the help of the Kolmogorov–Smirnov test. The numerical variables of the study groups were compared using Student's *t*-test in case of normally distributed data. On the other hand, the Mann–Whitney *U* test was used in case of abnormally-distributed data, both in case of independent samples. In order to compare the categorical data, the authors used the Chi-square (*χ*^2^) test. In case when the expected frequency is less than 5, the exact test is applied. *p* value <5 was considered statistically significant. The entire set of statistical analyses was conducted using MS Office Excel 2019 (Microsoft Corporation, NY, USA) and IBM SPSS (Statistical Package for the Social Science; IBM Corp, Armonk, NY, USA) release 22 for Microsoft Windows.

## 3. Results

**Figure 1:**
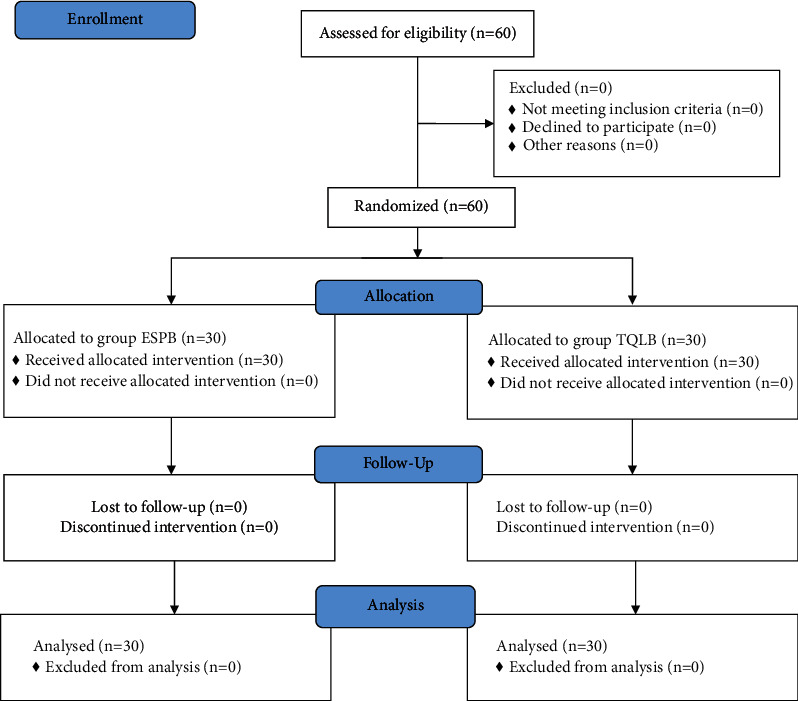
CONSORT flow diagram.

**Figure 2:**
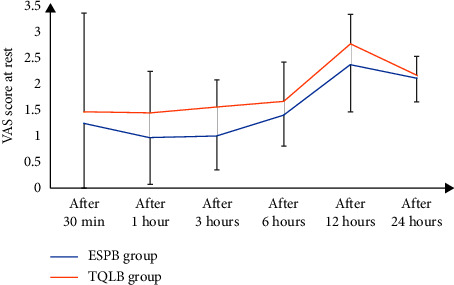
Mean VAS score at rest between the two groups over the study period.

**Figure 3:**
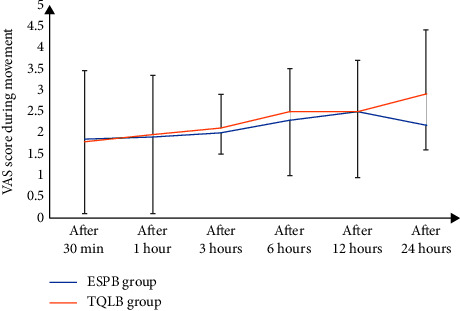
Mean VAS score during movement between the two groups over the study period.

**Figure 4:**
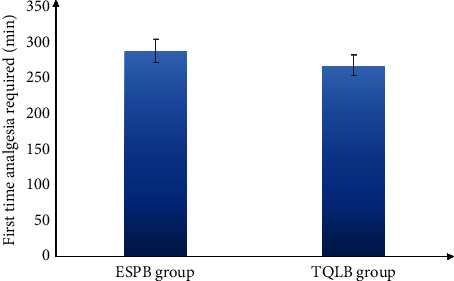
Mean time to 1^st^ analgesic request (min) between the 2 study groups.

**Figure 5:**
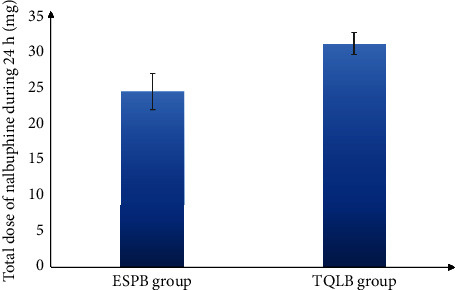
Mean total nalbuphine consumption (mg) between the 2 study groups during 24 h.

As shown in the CONSORT flow diagram (Figure 1), 60 participants underwent initial screening and were randomly assigned to two groups of 30 each. None of the study subjects withdrew from the study. Both demographic data as well as the operative features of the respondents are shown in (Table 1). Both groups exhibited no remarkable difference in terms of mean age, BMI, or ASA physical status. In concordance with that, the intraoperative variables of the time taken for surgery, anesthesia, and PACU stay were compared between the groups. The time required to perform the technique was found to be remarkably less in the ESPB group than in the TQLB group (*p* < 0.001). According to the VAS scores at rest during different time intervals, there was no significant difference found between the groups at 30 min, 6 h, and 24 h at rest after surgery (*p* > 0.05). Nevertheless, the VAS scores significantly differed at 1, 3, and 12 hours after surgery between the groups, i.e., were lower in the ESPB group than the TQLB group (*p* < 0.05) (Table 2 and Figure 2). In addition, no significant difference was found in the VAS scores during the movement between the study groups at 30 min and 1, 3, 6, and 12 hours after surgery (*p* > 0.05). Nonetheless, the VAS score was secured by the ESPB group at 24 hours after the surgery during movement than the TQLB group (*p* < 0.05), as shown in Table 2 and Figure 3. The time for raising the request for the first rescue analgesic was found to be remarkably longer in the ESPB group (280 ± 15.5 min) than in the TQLB group (260 ± 13.8 min) (Table 2 and Figure 4). Likewise, the overall nalbuphine consumption during the first 24 hours was found to be remarkably lower in the ESPB group (24 ± 2.5 mg) than the TQLB group (30.5 ± 1.55 mg) (Table 2 and Figure 5). Both groups exhibited no remarkable difference in terms of QoR-15 scores at the preoperative (physical well-being and mental well-being) stage (*p*=0.924), while at 24 hours, the ESPB group was found to be significantly better than the TQLB group (*p* < 0.001) (Table 3). When determining patient satisfaction with pain relief during 24 hours, the patient satisfaction in the ESPB group was found to be significantly better than that of the patients in the TQLB group (*p* < 0.001) (Table 3). There was no significant difference found between the groups in terms of postoperative hypotension (*p*=0.531) or postoperative vomiting (*p*=0.704) (Table 4). None of the patients suffered from respiratory depression or sedation in both groups (Table 4).

## 4. Discussion

The current study contrasted the impact of bilateral ultrasound-guided ESPB and TQLB upon providing relief to patients suffering from postoperative pain who underwent elective laparoscopic surgery for CRC. According to the VAS scores at rest, a significant difference was found between the groups at 1, 3, and 12 hours after surgery, i.e., the ESPB group secured lower VAS scores than the TQLB group. At 24 hours after the surgery, the ESPB group secured lower VAS scores during movement than the TQLB group. In addition to these, the ESPB group took a longer time to raise their first request for rescue analgesics compared to the TQLB group. Likewise, the overall nalbuphine consumption during the first 24 hours was remarkably low in the case of the ESPB group compared to its counterpart. In terms of QoR-15 scores at 24 hours, the ESPB group was significantly better than the TQLB group. However, no significant difference was found between the groups in terms of postoperative hypotension or postoperative vomiting.

Although the pain is comparatively less in laparoscopic surgery than in laparotomy, the former procedure is not a completely pain-free one. In laparoscopy, the pain is related to damaged blood vessels, release of inflammatory substances as a result of peritoneal distension, and the occurrence of nerve traction [24]. During the laparoscopic procedure for CRC, the analgesia must block the visceral pain, while at least T8-L2 abdominal dermatomes should be blocked during this procedure since the incisions are mostly paraumbilical or subumbilical. Various approaches of QLB reached the sensory levels of T7-L1 dermatomes [25]. As per the findings of Hansen et al. [26], the application of ultrasound-guided QLB in caesarean section predominantly mitigates the consumption of opioids after the procedure, and it also enhances the time taken to raise the first request for opioids. Kwak et al. [27] mentioned that preoperative unilateral QLB mitigated the postoperative pain successfully and reduced the consumption of opioids after laparoscopic nephrectomy. From the meta-analysis results [28], it can be inferred that QLB can be applied in the case of abdominal or hip surgery patients since it has the potential to mitigate the intensity of the pain and the consumption level of opioids after surgery. Wang et al. [29] identified that the lateral QLB procedure can improve the recovery process after laparoscopic CRC with an enhanced QoR-15 score after 48 hours of the operative procedure. On the contrary, Kawk et al. [27] argued that lateral QLB fails to enhance the global QoR-15 score after 48 hours of surgical procedure among laparoscopic nephrectomy patients. This inference would have been achieved as a result of not analysing the efficacy of QLB block after 24 hours of the surgical procedures. In a review conducted among 2,382 patients handled with QLB block (771: lateral approach; 1485: posterior approach; and 81: anterior approach), Ueshima and Hiroshi [30] identified the incidence rate of quadriceps muscle weakness in these three approaches to be 1%, 19%, and 65% correspondingly. Among these approaches, the anterior approach had the highest incidence rate.

The underlying mechanisms behind ESPB and paravertebral block were assumed to be similar since both tend to have multidermatomal sensory blocks of the posterior, lateral, and anterior thoracic wall [8]. A study has proved the penetration of the epidural space, neural foramen, and intercostal spaces when the dye was deeply injected into erector spinae (ES) muscles in cadavers [31]. This confirms the proposed mechanism of ESP block to achieve visceral and somatic sensory blocks [8]. In general, during ESP block, local anaesthesia is applied between the transverse process of the vertebra and the erector spinae muscle fascia. Once the injection is administered, the local anesthetic spreads caudally and cranially, thus creating an impact on the wide dermatomal area. The purpose of ESPB during abdominal surgery is to ensure somatic and visceral analgesia by impacting the ventral rami of the spinal nerves [8, 32].

When ESPB is performed at T10, it has the potential to trigger sensory loss from T5 to L2 [33]. In the literature, the magnetic resonance imaging (MRI) procedure was used to decode that the ESPB mechanism is effective due to its spread into the epidural and transformational space. This provides an advantage to ESPB compared to the rest of the interfascial plane blocks of the thorax, such as transversus abdominis plane block (TAPB), pectoralis nerve block, and serratus anterior. Unlike these methods, abdominal visceral analgesia is also rendered by the ESP block [34]. According to Choi et al. [35], after the induction of anesthesia, bilateral ESPB reduces the requirement for opioids and mitigates the pain after surgery among laparoscopic CRC patients. However, the first postoperative day of these patients showed no significant improvement in their QoR. According to Qi-hong et al. [36], ESPB is a highly efficient regional block technique that can be used to provide relief from postoperative pain to aged patients who are undergoing laparoscopic CRC than oblique subcostal TAPB. When applying bilateral ESPB in laparoscopic cholecystectomy and bariatric surgery at the T7 level as a means of analgesic after the operative procedure, there was a decline observed in the visceral pain, somatic abdominal pain, and the VAS score [37, 38]. In enhanced recovery after surgery (ERAS) protocol, it is important to have an effective postoperative pain relief measure. So, the facial plane blocks must be supplemented with the multimodal analgesia regimen [39]. After the CRC surgery is completed [40], about 25% of the patients suffer from postoperative ileus. Furthermore, the delay in the optimization of bowel function remains a crucial reason behind the prolonged hospital stay of the patients [41]. In ERAS protocols, a few crucial components are mentioned, including the mitigation of patient discomfort and the reduction of hospital stay period by preventing the ileus. As per the literature [42, 43], the reduction in the consumption of opioids has a positive association with increased recovery quality and shorter mobilization time.

According to Jiang et al. [44], both ESPB and TQLB procedures enhance the quality of multimodal analgesia among patients undergoing total laparoscopic hysterectomy (TLH). In addition to these, after TLH, ESPB is one such preferred interfascial plane block technique since it remarkably reduces the visceral pain after the operative procedure and lessens the consumption of opioids. According to Kang et al. [45], bilateral single-injection QLB fails to mitigate the consumption of cumulative opioid levels during 24 hours of postoperative time compared to bilateral single-injection ESPB in patients undergoing laparoscopic liver resection. On the other hand, Aksu et al. [46] mentioned that ESPB has the ability to generate postoperative analgesic effect in paediatric patients, alike the QLB, who are undergoing lower abdominal surgery. According to the researchers, ESPB is a comparatively safer method, whereas the associated complications and risks are lower. Only a handful of studies exist to report the complications triggered by ESPB, in which two studies cited about pneumothorax associated with ESPB [47, 48]. This low incidence of complications might be attributed to the place of injection being far from the pleura and major vessels.

The current study has a few limitations to overcome. In spite of calculating the sample size, the number of patients included in the study was too small to generalize the study outcomes. So, future studies must be conducted with a large number of samples. Second, the blocks were performed after inducing the anesthesia while the patients were unconscious. So, in this scenario, the blockage level or its strength cannot be confirmed since the purpose of the study was limited to analysing the effects of the block upon pain and the need for analgesia and not upon the distribution level of the sensory blocks. Third, the patients were followed only for a shorter period of time, i.e., only 24 hours. At the end, the study did not measure the impact of the blocks upon time to ambulation or the duration of the hospital stay.

## 5. Conclusion

Bilateral ultrasound-guided ESPB took a considerably longer time to raise the first request for rescue analgesics in patients undergoing laparoscopic resection of colorectal cancer than the bilateral ultrasound-guided TQLB. Moreover, ESPB significantly reduced the overall opioid consumption during the first 24 hours compared to the TQLB procedure. No significant difference was found between the blocks in terms of postoperative complications.

## Figures and Tables

**Table 1:** Demographic data and operative characteristics.

	ESPB (*n* = 30)	TQLB (*n* = 30)	*p* value
Age (years)	50 ± 7.29	53 ± 8.11	0.137
Sex (males/females)	17/13	11/19	0.121
BMI (kg/m^2^)	24.66 ± 3.8	24.56 ± 3.5	0.916
ASA physical status (I/II/III)	4/23/3	9/20/1	0.209
Time needed to perform the technique (min)	4 ± 0.6	6.2 ± 0.9	<0.001
Duration of surgery (min)	144 ± 42	150 ± 60	0.655
Duration of anesthesia (min)	186 ± 48	198 ± 72	0.424
Duration of PACU stay (min)	58.6 ± 9.6	58.2 ± 9.3	0.870

Data are presented as the mean ± standard deviation (SD) or as the number of patients. *p* value <0.05: significant, *p* value >0.05 nonsignificant.

**Table 2:** VAS score between the 2 groups at rest and movement, time to 1^st^ rescue analgesia (minutes), and total dose of nalbuphine during the 1^st^ 24 h (mg).

	ESPB (*n* = 30)		TQLB (*n* = 30)	*p* value
*VAS score during rest*				
After 30 min	1.24 ± 1.65		1.45 ± 1.88	0.647
After 1 hour	0.96 ± 0.88		1.44 ± 0.78	0.029
After 3 hours	1 ± 0.65		1.55 ± 0.51	0.001
After 6 hours	1.4 ± 0.6		1.65 ± 0.75	0.159
After 12 hours	2.35 ± 0.9		2.75 ± 0.55	0.042
After 24 hours	2.1 ± 0.45		2.15 ± 0.35	0.633

*VAS score during movement*				
After 30 min	1.85 ± 1.75		1.8 ± 1.65	0.910
After 1 hour	1.9 ± 1.8		1.95 ± 1.4	0.905
After 3 hours	2 ± 0.5		2.1 ± 0.8	0.564
After 6 hours	2.3 ± 1.3		2.5 ± 1	0.507
After 12 hours	2.5 ± 1.55		2.5 ± 1.2	1.000
After 24 hours	2.2 ± 0.6		2.9 ± 1.5	0.021

First time analgesia required (min)		280 ± 15.5	260 ± 13.8	<0.001
Total dose of nalbuphine during 24 h (mg)		24 ± 2.5	30.5 ± 1.55	<0.001

Data are presented as the mean ± standard deviation (SD). *p* value <0.05: significant, *p* value >0.05: nonsignificant.

**Table 3:** QoR and patient satisfaction.

	ESPB (*n* = 30)	TQLB (*n* = 30)	*p* value
QoR-15 score at preoperative	148.6 ± 12.3	148.7 ± 11.8	0.924
Physical well-being	89.2 ± 7.1	89.4 ± 6.5	0.910
Mental well-being	59.4 ± 5.2	59.3 ± 3	0.928
QoR-15 score at 24 hours	124.4 ± 12.5	109.7 ± 14.6	<0.001
Physical well-being	75.1 ± 4.2	67.3 ± 8.1	<0.001
Mental well-being	49.3 ± 8.3	42.4 ± 6.5	0.001
Patient satisfaction during the 1st 24 h (poor/moderate/good/excellent)	0/3/20/7	3/15/11/1	<0.001

Data are presented as the mean ± standard deviation (SD) or as the number of patients. *p* value <0.05: significant, *p* value >0.05: nonsignificant.

**Table 4:** Postoperative complications.

	ESPB	TQLB	*p* value
Hypotension	5	8	0.531
Bradycardia	0	0	—
Respiratory depression	0	0	—
Sedation	0	0	—
Vomiting	3	5	0.704

Data are represented as the number of patients. *p* value <0.05: significant, *p* value >0.05: nonsignificant.

## Data Availability

The datasets used and analysed during the current study are available from the corresponding author upon reasonable request.
